# Supplementary material for: Superconductor-insulator transition in space charge doped one unit cell Bi2.1Sr1.9CaCu2O8+x
Source: Nat Commun. 2021 May 18;12:2926. doi: 10.1038/s41467-021-23183-z (PMC8131387; doi:10.1038/s41467-021-23183-z)
Supplement: Supplementary file 1 — Supplementary Information [file 41467_2021_23183_MOESM1_ESM.pdf]

SUPPLEMENTARY INFORMATION

Superconductor-Insulator Transition in space charge doped one unit cell $Bi_{2.1}Sr_{1.9}CaCu_2O_{8+x}$

Fang Wang,[1] Johan Biscaras,[1,*] Andreas Erb,[2] and Abhay Shukla[1,†]

[1]*Sorbonne Université, CNRS UMR7590, MNHN, Institut de Minéralogie, de Physique des Matériaux et de Cosmochimie, IMPMC, 75005 Paris, France*

[2]*Walther Meissner Institut fur Tieftemperaturforschung, Bayerische Akademie der Wissenschaften, Walther-Meissnerstr. 8, D-85748 Garching Germany*

SUPPLEMENTARY NOTE 1: DOPING REVERSIBILITY

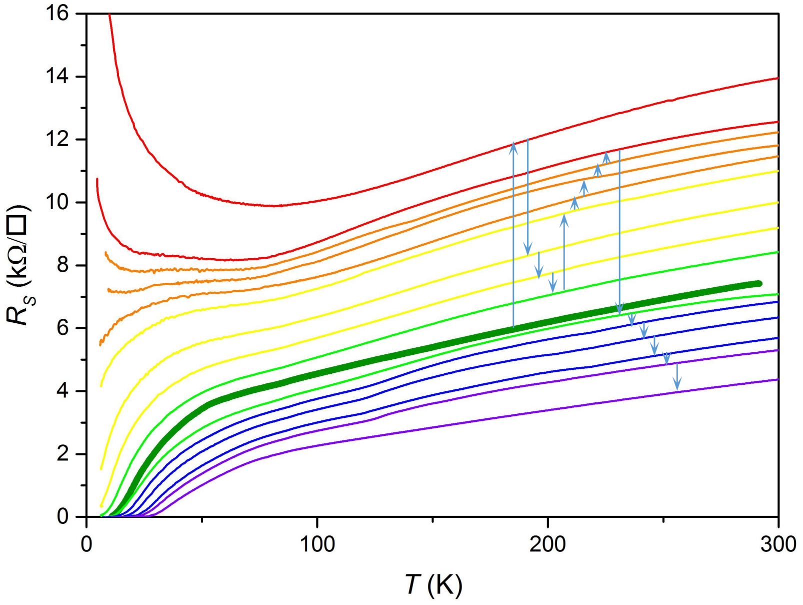

Supplementary Figure 1. **Doping reversibility.** Temperature dependence of sheet resistance driven by space charge doping method on a linear scale. The thick olive $R_S(T)$ curve indicates the starting doping level after annealing in the oven. The left-to-right direction of the arrows shows the order of doping step by step, which shows the reversibility of this electrostatic doping method. The temperature for doping varies from 350 K to 380 K. A positive (or negative) voltage, applied to the back gate of the device (main text Fig. 1**a**), decreases (or increases) the doping level. The value of the voltage varies from 100 V to 280 V and the corresponding doping time varies from 10 minutes to 110 minutes according to the initial and final doping values.

SUPPLEMENTARY NOTE 2: SCALING ANALYSIS

For the scaling analysis between 9 K and 20 K, at each given temperature, all the sheet resistance data from Fig. 1**b** in the main text were firstly plotted as a function of doping p (Fig. 4**a** of main text). The way of the determination of doping level p is detailedly explained in the main text. This procedure results to a crossing point $p_c \approx 0.05747$, and the corresponding critical sheet resistance $R_c \approx 6.85$ kΩ.$\square^{-1}$ (Fig. 4**a** of main text). Then by changing the scaling abscissa from p to $|p - p_c|t$, where t is a scaling factor that we have varied at each temperature to maximize collapse of each isothermal sheet resistance curve, all the isothermal curves collapse onto the single finite-size scaling function $R_S = R_c f(|x - x_c|T^{-1/vz})$, where x is the doping level p in our case. The collapse fits perfectly up to 15 K (Fig. 4**b** of main text). Finally according to the power law, by plotting T as a function of t on a log-log scale, the critical exponent vz, extracted from the value of the slope of the curve $T(t)$ (Inset Fig. 4**b** of main text), equals to 1.57 ± 0.01.

Supplementary Figure 2 shows the finite-size scaling between 7 K and 20 K. This scaling procedure is exactly the same as that between 9 K and 20 K. In Fig. 2**a** of supplementary, all sheet resistance collapse more perfectly onto a single function, which shows that critical sheet resistance $R_c = 6.85 \pm 0.1$ kΩ.$\square^{-1}$. Then we also plot the relation between T and t between 7 K and 20 K on a double logarithmic scale, as shown in Fig. 2**b** of supplementary. Deviations from linearity are found at extremes. The possible reason can be thermal fluctuations at high temperature and defect related to weak localization at low temperature.

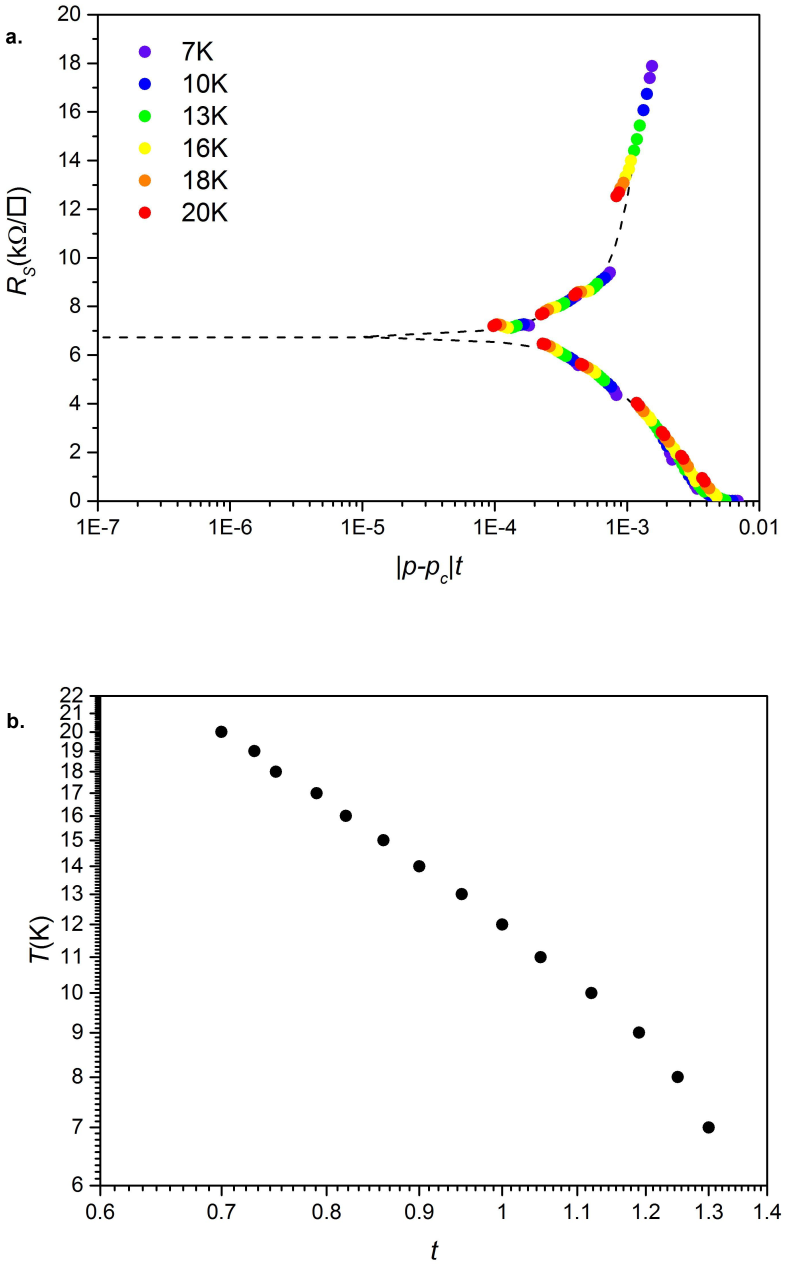

Supplementary Figure 2. **Scaling analysis between 7 K and 20 K. a** Scaling of the same sheet resistance data as in Fig. 4**a** of main text as a function of $|p - p_c|t$ with $t = T^{-1/vz}$. **b** The linear relation between T and t from 7 K to 20 K on a double logarithmic scale.

* johan.biscaras@sorbonne-universite.fr
† abhay.shukla@sorbonne-universite.fr
